# COVID-19 Surveillance in the Primary Health Care Population of Qatar: Experience of Prioritizing Timeliness Over Representativeness When Sampling the Population

**DOI:** 10.3389/fpubh.2021.654734

**Published:** 2021-05-07

**Authors:** Hamda Abdulla A/Qotba, Ahmed Sameer Al Nuaimi, Hanan Al Mujalli, Abduljaleel Abdullatif Zainel, Hanan Khudadad, Tamara Marji, Shajitha Thekke Veettil, Mohamed Ahmed Syed

**Affiliations:** ^1^Department of Clinical Research, Primary Health Care Corporation, Doha, Qatar; ^2^Directorate of Clinical Affairs, Primary Health Care Corporation, Doha, Qatar

**Keywords:** COVID-19, surveillance, sampling methods, quality, primary health care

## Abstract

SARS-CoV2 a new emerging Corona Virus Disease in humans, which called for containment measures by many countries. The current paper aims to discuss the impact of two different sampling methodologies when executing a drive through COVID-19 survey on the quality of estimated disease burden measures. Secondary data analysis of a pilot cross-sectional survey targeting Qatar's primary health care registered population was done. Two groups with different sampling methods were compared for estimating COVID-19 point prevalence using molecular testing for nasopharyngeal swabs. The first group is a stratified random sample non-proportional to size (*N* = 260). A total of 16 population strata based on age group, gender, and nationality were sampled. The second group is the Open invitation group (*N* = 841). The results showed that the two groups were obviously and significantly different in age and nationality. Besides, reporting of COVID-19 symptoms was more frequent in the open invitation group (28.2%) than the random sample (16.2%). The open invitation group overestimated the symptomatic COVID-19 prevalence rate by more than four times, while it overestimated the asymptomatic COVID-19 cases by a small margin. The overall prevalence rate of active COVID-19 cases in the open invitation sample (13.3%) was almost double that of the random sample (6.9%). Furthermore, using population sampling weights reduced the prevalence rate to 0.8%. The lesson learned here is that it is wise to consider the magnitude of bias introduced in a surveillance system when relying on convenient sampling approaches in response to time constraints.

## Introduction

On 31st December 2019, Chinese national authorities reported an outbreak of pneumonia with unknown etiology (1). On the 12th of January 2020, National Health Commission in China associated the outbreak to a seafood market in in Wuhan (China) and shared the genetic sequence of the novel causative agent - a novel coronavirus (1).

Coronaviruses in the recent past have come to attention as pathogens of emerging respiratory disease outbreaks such as, Severe Acute Respiratory Syndrome (SARS) in 2002–3 and Middle East Respiratory Syndrome (MERS) in 2012–14. The newly identified coronavirus with its epicenter in Wuhan was labeled Severe Acute Respiratory Coronavirus 2 (SARS-CoV2) and is also known as 2019 novel coronavirus (2019-nCoV) and coronavirus disease 2019 (COVID-2019) (2).

SARS-CoV2 very quickly spread to other parts of China and the world. First imported cases were reported in Japan, Thailand and Republic of Korea between the 13–20th January (1). The first 1,000 cases were infected within 48 days a significantly higher rate compared to SARS and MERS which took 4 months and 2 and a half years, respectively (3). With 18 countries affected and as the outbreak continued to spread globally, the World Health Organization (WHO) declared it a Public Health Emergency of International Concern (PHEIC) on the 30th January 2020 (4). Eventually on March 11, 2020, the WHO declared the SARS-CoV2 outbreak a pandemic (5). Controlling the disease is still a priority worldwide with more than 116 million cases and 2,700 thousand deaths recorded until the 7th of March 2021 (6).

Primary care is the cornerstone of any health system. During pandemics, primary care is the frontline against emerging infectious diseases in communities. It provides infrastructure and plays a variety of key roles such as disease surveillance, diagnosis and treatment, prevention, patient education etc., (7). During the peak week of a pandemic, one can expect additional primary care visits (8). These present challenges and opportunities in primary care as the SARS-CoV2 continues to spread in the country. Among them is describing the extent of disease spread and population sectors most affected. Survey tools are needed to assess the disease burden (9). Such tools are subject to known, or at least anticipated to have biases which can threaten clinical and epidemiological studies (10).

In May 2020 the only available laboratory testing approach to screen for COVID-19 was using anasopharyngeal swab to analyze by reversed transcription polymerase chain reaction (rt-PCR). This laboratory approach was used to calculate a crude measure of population prevalence which is the fraction of positive tests in a cross-sectional time frame. Such a measure of disease frequency is always liable to distortion by ascertainment bias since tests are typically only ordered from symptomatic cases seeking health care, whereas, a large proportion of infected might show little to no symptoms. Contact tracing may reduce this distortion, but this will always depend on test availability and the capacity of surveillance health system (11, 12). It has been suggested that this capacity for rapidly identifying individuals infected with the virus can become more efficient by pooling (or combining) individual samples (30 to 100 samples) and testing them in a single group. Such a method can decrease the cost of screening contacts at the expense of reduced test sensitivity (13).

Sampling technique is the most important concept in survey studies, since it is impractical, uneconomical or feasible to test the whole population, even after considering pooling of individual samples as a method of cost reduction. The sample should represent the population for the survey results to have external validity. It is clear that random samples are superior to convenient ones for quantitative research studies. However, a pandemic like COVID-19 may call for desperate actions and serves as an excuse for using less stringent criteria in choosing survey samples without assessing the extent of bias introduced during the process (14). Containment measures may push for an expedited approach to epidemiologic info.

A survey was designed to estimate prevalence in Qatar's primary care registered population (15).The aim of this paper is to present the lessons learned from using two different sampling methodologies applied when executing the survey. In addition, it provides a snapshot of the COVID-19 outbreak in Qatar's primary care registered population after 3 months from the start of the COVID-19 pandemic.

## Methods

The current study is based on secondary data analysis for a two days cross-sectional pilot survey study executed on May 2020. The study protocol for the survey was designed by the Department of Clinical Research at Primary Health Care Corporation to generate epidemiologic data to plan and respond to the pandemic in Qatar.

### Study Settings

Qatar, a peninsular Arab country that operates a universal publicly funded health care system accessible to Qatari national and expatriates who hold a valid health card. The primary healthcare service in Qatar are delivered by the Primary Health Care Corporation (PHCC), which is the largest primary care provider in the country with 27 health centers distributed across three geographical regions – North, Central and South.

### Study Samples

The survey originally targeted a random sample of PHCC registered population (*N* = 1,063,243 as of May 2020 or ~70% of the total population of Qatar) with only two working days assigned for data collection phase. This group is referred to as the “Random Sample Group” (RSG). The sampling method was a stratified random sample non-proportional to size. The stratifying factors were age group, gender and nationality representative of the overall PHCC registered population. A total of 130 individuals were randomly selected from each of 16 population strata. To adjust for non-response, 50% extra participants were added (*n* = 65). The final strata sample size will be 195 and the resulting total sample size will be 3,120.This sampling approach was used to ensure adequate representation for all population strata, while obtaining a representative summary measure for the reference population through proper weighting at the analysis stage. The details of the survey protocol are published elsewhere (15).

During the first day of the data collection phase of the survey a low response rate of around 10% was observed and a decision was made to send an open invitation to all the PHCC registered population to attend on the second (last) day of the pilot survey. The Open Invitation Group (OIG) was recruited during the second and final day of the survey. SMS messages were sent to every individual in the target population (PHCC registered population) providing them with the opportunity to be tested for COVID-19 on the next day if they register themselves on a designated web site.

### Study Locations

One PHCC health center from each of the three regions in Qatar were identified as a study location - Al Thumama (South), Leaibab, and Al Waab. The health centers were set up to facilitate drive through testing of participants. This setup of test locations allowed equal chances for the invited residents from each of the three principal regions of Qatar to access them.

### Invitation

The study was conducted over 2 days (5th and 6th of May). A national campaign to publicize the study was initiated 2 days prior its launch using social media and newspapers. RSG Participants were also sent an SMS message inviting them 2 days in advance. The SMS message included a link to a questionnaire survey to accept or decline the invitation. All participants were invited to attend a study location in the same region as the health center they were originally registered.

### Data Collection

Data collection at study locations was undertaken as a drive through. Participants were seated in their cars and queued to be attended by a data collector. Data collection was undertaken as a 4-step process, steps 1–3 by a data collector and step 4 by a trained nurse.

- Step 1: Verify participants' identification details.
- Step 2: Confirm participant was invited by SMS or not.
- Step 3: Administer a questionnaire to collect information on their age, gender, nationality, and COVID-19 symptoms.
- Step 4: Provide a nasopharyngeal swab.

### Laboratory rtPCR Test Procedure

The nasal and throat swabs were labeled and transported from the study location to the referral laboratory for the state of Qatar's at the end of each shift. RNA was extracted and isolated prior to amplification using the rtPCR (reverse transcription polymerase chain reaction) test. Each assay was validated for cycle threshold (CT) value interpretation using the manufacturer's instructions. Test results were reported as negative or positive (16).

### Data Analysis

All data was subject to quality assurance. For the purposes of this study, point prevalence was defined as the number of active SARS-CoV2 infections (identified by RT-PCR) over the total sample size. Chi-square test of independence was used to assess the statistical significance of associations between nominal or ordinal scale variables. *P*-value less than the 0.05 level of significance was considered statistically significant. All statistical analyses were done using survey commands in SSPS (version 23).

Sampling weights are the inverse of the likelihood of being sampled. The purposes of weighting the summary prevalence estimate of the population at the analysis stage was to compensate for non-response and the unequal probabilities of selection. The sampling fraction and the response rates in each population strata were used as sampling weights (17). Please refer to Supplementary Material for further details of calculations.

### Positive Test Results

All study participates were informed of their test results by SMS. All the participants who tested positive for SARS-CoV-2 were contacted by telephone by a team designated by the authorities to track their infection.

### Ethical Considerations

The current study is based on anonymized secondary data analysis of a pilot survey study executed on the 4th and 5th of May 2020. The study presented a minimal risk of harm to its subjects since there was no direct interaction with study participants and the data was requested from the data custodian in PHCC with no personal identifiers. Overall, the study was conducted with integrity according to generally accepted ethical principles and was approved by the PHCC's independent ethics committee (PHCC/DCR/2020/05/051).

## Results

The results presented in this section were based on the analysis of 841 individual in the open invitation group and 260 individual in the random sample group. As shown in Table 1, there was an obvious and statistically significant difference in age distribution between the two study groups. The random sample being older in age than the open invitation. Gender distribution was however not different with females constituting less than one fifth of the two study groups. The composition of the groups according to nationality was also significantly different. Qataris and other Arab localities being less represented in the open invitation group, while Southern Asia and South-Eastern Asia nationalities were over-represented in the same group compared to random samples.

**Table 1:** Comparing the two study samples by sociodemographic variables.

	**Open invitation group**	**Random sample group**	***P***
	***N* (%)**	***N* (%)**	
**Age group (years)**			<0.001
<18	25 (3.0)	0 (0.0)	
18–39	579 (68.8)	123 (47.3)	
40–59	216 (25.7)	106 (40.8)	
60–74	21 (2.5)	31 (11.9)	
Total	841	260	
**Gender**			0.91 [NS]
Female	161 (19.1)	49 (18.8)	
Male	680 (80.9)	211 (81.2)	
Total	841	260	
**Nationality**			<0.001
Qatar	110 (13.1)	56 (21.5)	
Other Arab countries	164 (19.5)	83 (31.9)	
Europe/North America/Australasia	21 (2.5)	11 (4.2)	
Southern Asia	456 (54.2)	96 (36.9)	
South-Eastern Asia	81 (9.6)	7 (2.7)	
Eastern-Central Asia	0 (0.0)	5 (1.9)	
Rest of Africa	9 (1.1)	2 (0.8)	
Total	841	260	

A history of contact with suspected or confirmed case in the last 2 weeks was significantly more frequent in the open invitation group (32%) compared to random sample group (13.3%). In addition, almost all the reported symptoms were more frequent in the open invitation group. Three of the symptoms, namely: fever 38°C or higher, sore throat and cough were significantly more frequent among the open invitation group compared to the random sample. The proportion of symptomatic subjects with at least one symptom in the last 2 weeks was also significantly higher in the open invitation group (28.2%) compared to random sample (16.2%), Table 2.

**Table 2:** The difference in relative frequency of selected symptoms between the two study groups.

**Symptoms/complaints in the last 2 weeks**	**Open invitation group (*****n*** **=** **841)**		**Random sample group (*****n*** **=** **260)**		***P***
	**N**	**%**	**N**	**%**	
Fever 38°C or higher	63	7.5	8	3.1	0.011
Sore throat	87	10.3	12	4.6	0.005
Cough	109	13.0	10	3.8	<0.001
Chills	4	0.5	1	0.4	1 [NS]
Fatigue	18	2.1	3	1.2	0.43 [NS]
Muscle ache	22	2.6	4	1.5	0.31 [NS]
Runny nose	34	4.0	9	3.5	0.67 [NS]
Shortness of breath	23	2.7	2	0.8	0.06 [NS]
Wheezing	5	0.6	2	0.8	0.67 [NS]
Chest pain	23	2.7	3	1.2	0.14 [NS]
Other respiratory symptoms	25	3.0	3	1.2	0.10 [NS]
Headache	68	8.1	12	4.6	0.06 [NS]
Nausea/vomiting	6	0.7	0	0.0	0.35 [NS]
Abdominal Pain	4	0.5	4	1.5	0.1 [NS]
Diarrhea	9	1.1	0	0.0	0.13 [NS]
Loss of sense of smell	15	1.8	1	0.4	0.14 [NS]
Loss of sense of taste	9	1.1	1	0.4	0.47 [NS]
At least one symptom (in the last 2 weeks)	237	28.2	42	16.2	<0.001
Complaints requiring medical attention	9	1.1	3	1.2	1 [NS]

**Figure 1:**
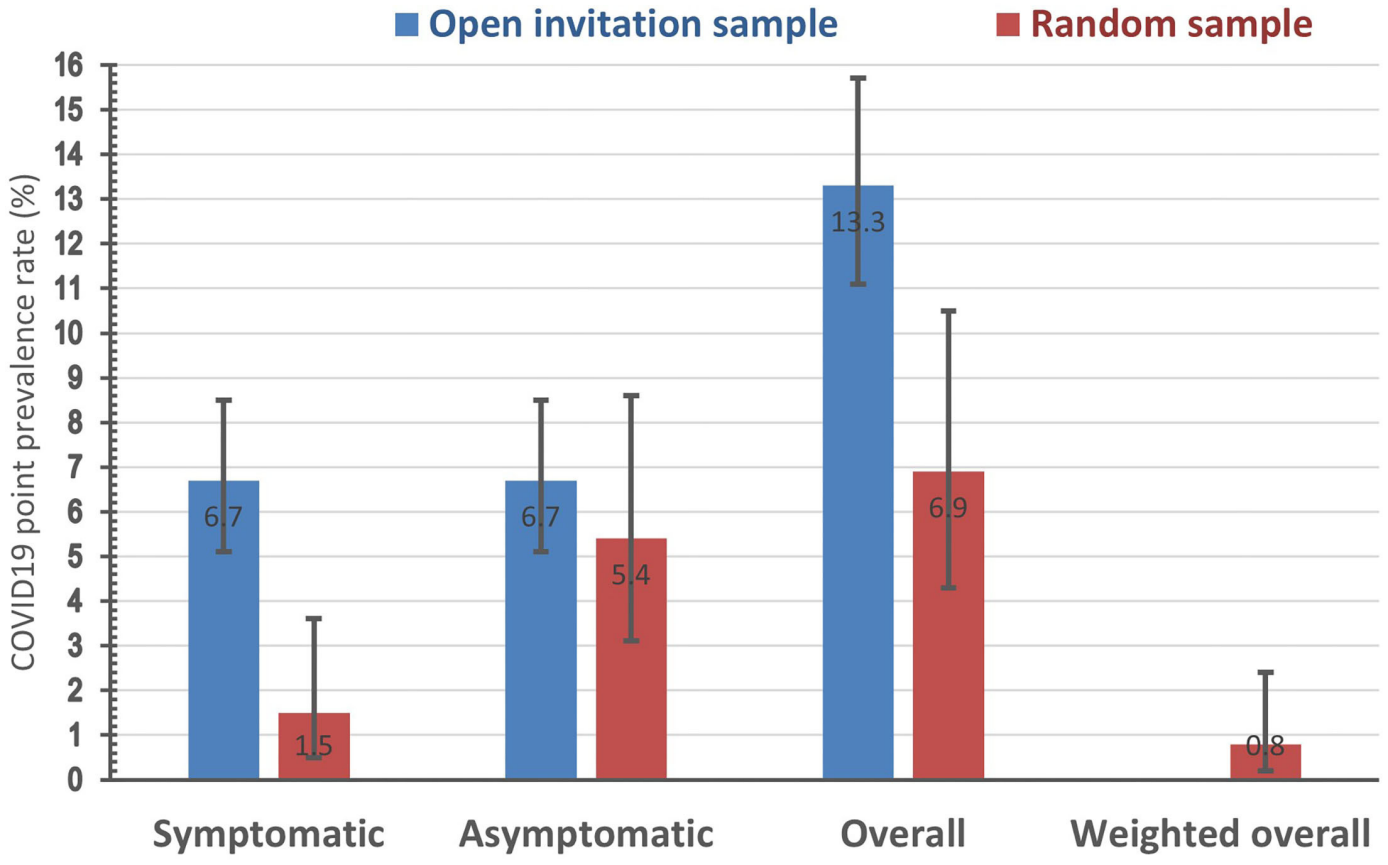
Point prevalence rate (with its 95% confidence interval) of positive rtPCR COVID-19 test in two study samples by symptoms status.

The prevalence rate of symptomatic COVID-19 cases was more than four times higher in the open invitation sample (6.7%) compared to the random one (1.5%). However, that of asymptomatic cases was only marginally higher in the open invitation sample (6.7%) compared to random one (5.4%). The overall prevalence rate of active COVID-19 cases in the open invitation sample (13.3%) was almost double that of the random sample (6.9%). The ratio of asymptomatic to symptomatic COVID-19 cases the random sample group was 3.6, while it was exactly 1 for the open invitation sample. The crude (unweighted) overall population prevalence rate in the random sample was 6.9%, while the weighted estimate after adjustment for the sampling fraction in of the 12 population strata available for analysis (the four strata of those younger than 18 years had a null value as none of these strata were respondents) is only 0.8% (with a 95% confidence interval ranging between 0.2 to 2.4%), Figure 1.

The cumulative prevalence rate of all positive COVID-19 PCR test from the time when the first case was recorded in Qatar on 29/2/2020 until the last day of the current study survey is 0.6%, Table 3.

**Table 3:** Estimated period prevalence rates of Qatar population.

	Confirmed cases (PCR positive)^*^	**PCR tests performed**	**Population size**	**Test yield (PCR test positivity rate)**	**Period prevalence estimate (%)**
Cumulative counts since the start of pandemic (29/2/2020) till the last day of the current survey (5/5/2020)	17,142	109,762	2,807,805	15.6	0.6

* *Note: These are national figures of COVID-19 positive PCR test results. They include the cases discovered in the current study survey*.

## Discussion

As the demand for accountability increased in the recent time the quality of data and reported figures has become crucial for public health program's performance. According to MEASURE Evaluation “data must be of high quality if they are to be relied upon to inform decisions on health policy, health programs, and allocation of scarce resources” (18). Among the important elements of data quality is relevance, accuracy, comparability, and timeliness (19). The first three of these elements can only be assured by using a random sample. In addition, using mathematical modeling to measure bias is an established method in research (12), but the current study is among few that provides an opportunity to measure it directly in a real life example comparing the results provided non-random sample (OIG) to the random one (RSG).

The COVID-19 survey was planned as a sentinel surveillance to be repeated at regular intervals on a representative batch of nasopharyngeal specimens, which is strongly advised by WHO as a strategy to identify and estimate community cases and inform planning especially in a primary care setting (20). A probabilistic sampling in a determined population is the method of sampling advocated that organization in the context of COVID-19. Disease positivity rates obtained from surveillance is subject to distortion with under-ascertainment of cases being the most important. This type of bias is especially disturbing in pandemics of new diseases with wide variation in clinical features as this can impact the implementation of public health policy and risk awareness (20). Interestingly, the current study showed an inverse type of bias affecting the surveillance system that was tested in primary health setting, that is an over-estimation of the point prevalence rate driven by the open invitation sample. This type of convenient non-probability sample was used in a COVID-19 population survey in Iceland, where a total of 10,797 persons received open invitations and another 2,283 invited in a random sample selection. The Icelandic study which was executed during March and April of 2020 showed that random sampling was associated with a lower proportion of positive PCR test results for COVID-19 (0.6%) compared to the open invitation group (0.8%) (21).

The current study in Qatar showed an obvious and statistically significant difference in age group and nationality representations between the random sample and open invitation group. A history of contact with suspected or confirmed case in the last 2 weeks, which is clearly an important risk factor for testing positive for COVID-19 was almost three times more frequent in the open invitation group. This difference may serve as an explanation for the overestimation bias caused by the open invitation group in a time where testing COVID-19 was not available for personal motives. Having a contact history motivates an individual to seek for COVID-19 testing and increase the probability of responding to an open invitation for testing COVID-19, since the test is not available upon personal request. The second argument that may serve as an explanation for the over-estimation bias caused by the open invitation group in the current study is the higher frequency of reporting fever, sore throat and cough among that group that motivated those individuals to favorably reply to the invitation sent. Similarly, this may give some clue to the equal ratio of asymptomatic and symptomatic COVID-19 cases detected in the open invitation group, while asymptomatic individuals constituted the majority in the random sample group.

The stratified random sample non-proportional to size was used in this study to facilitate logistics required for a short study period, therefore a weighted summary estimate of the point prevalence rate was calculated which further reduced the prevalence rate from 13.3% in the open invitation group to 6.9% in the crude unweighted random sample to only 0.8% in the weight random sample estimate. This weighted prevalence estimate of active COVID-19 cases (defined at that time as any individual with a positive COVID-19 PCR test) in the current survey is still bigger than the 0.6% population period prevalence rate covering the 2 months period of COVID-19 from its first reported case on 29/2/2020 till the last day of the current survey. One can argue that this difference is expected to be larger after considering the underestimation bias possibly introduced by the small response rate in the 0.8% prevalence figure and the overestimation bias in the period prevalence introduced by including some cases that are currently recovered.

The current study has its own limitations also. The random sample group represented a response rate of <10% for the targeted sample size. This was actually the reason behind opting to include an open invitation group. The strata of children 10–17 years old was completely missing from the random sample. Its worth noting that only two sample strata out of the total 12 available in the random sample showed positive COVID cases. These were Non-Qatari Males aged 18–39 years and 40–59 years. All the remaining strata showed no positive COVID-19 cases. These two strata had a higher opportunity for detecting positive cases, because they contained more tested people (they accounted for 62% of the completed random sample size of 260). One possible explanation for this finding is that COVID-19 is still localized in certain population subgroups and not widespread in the community at the time of executing the pilot survey. However, the high non-response rate in the random sample and the small sample size might bias such a conclusion. In addition, the COVID-19 cases included in the calculation of prevalence estimates were only those diagnosed using the random sample or the open invitation group during the 2 days of survey activities. The daily reported COVID-19 cases that present themselves to the health system or are captured by case finding screening activities are not part of the figures reported in this manuscript. Another possible source of bias introduced in the current survey is the effect of the infectious disease clustering in selected residential areas, which may have affected even the random sample because of the large non-response rate.

## Conclusion

The current study emphasized the importance for a robust sampling method in survey studies and the huge implications of sampling methodology on calculating COVID-19 prevalence estimates, which can inform critical decisions.

## Data Availability Statement

The data that support the findings of this study were requested from PHCC (Data custodian). As per PHCC rules that protect patient's privacy and confidentiality and the Confidentiality Agreement signed by the study team with PHCC the database can not be shared with external entities and can not be publicly available. Requests to access these datasets should be directed to researchsection@phcc.gov.qa.

## Author Contributions

HA/Q, MS, and HA: assisted in developing the study proposal and wrote parts of the first draft. In addition to reviewing the final version of the submission. AA: assisted in developing the study proposal, performed statistical analysis and wrote the study results section and discussion, and in addition to reviewing the final version of the submission. AZ, HK, TM, and SV: assisted in developing the study proposal and reviewed the final version of the submission. All authors contributed to the article and approved the submitted version.

## Conflict of Interest

The authors declare that the research was conducted in the absence of any commercial or financial relationships that could be construed as a potential conflict of interest.
