# Supplementary material for: COVID-19 Surveillance in the Primary Health Care Population of Qatar: Experience of Prioritizing Timeliness Over Representativeness When Sampling the Population
Source: Front Public Health. 2021 May 7;9:654734. doi: 10.3389/fpubh.2021.654734 (PMC8138580; doi:10.3389/fpubh.2021.654734)
Supplement: Supplementary file 1 [file Table_1.DOCX]

## Sampling method

Stratified random sample non-proportional to size. This method was favored over the stratified random sample proportional to size due to logistic constraints. Having too short time to target variable sample size strata in an accurate way. The research team opted to target fixed size strata to achieve adequate representation of each strata and postponing the weighting to the analysis stage to calculate an outcome (PCR positivity rate) that represents the primary health care population in a representative way. This method in addition, will allow for having a variable sample size in each strata after completing the survey, while not affecting the representativeness of the overall positivity rate estimate after using population weights in the analysis stage.

Three stratifying sociodemographic variables were chosen, namely: age group, gender and nationality. A total of 16 population strata were made by nesting the three categorical sociodemographic variables according to the number of categories for each, table 1.

Table 1: A description of the three sociodemographic stratifying variables.

| **Variable** | **Value (categories)** |
| --- | --- |
| Age group (years) | 60+ |
|  | 40-59 |
|  | 18-39 |
|  | 10-17 |
|  |  |
| Gender | Female |
|  | Male |
|  |  |
| Nationality | EXPATRIATE |
|  | QATARI |

To achieve a targeted sample size of 2080, which defines the total count of swabs allocated for this survey, a total of 130 individuals will be randomly selected from each stratum. Adding an extra 50% to each stratum sampled with adjust for the expected non-response rate. The final strata sample size is 195 and the resulting total sample size is 3120, table 2.

Table 2: Sample size per population strata

|  | **Per strata** | **Total sample** |
| --- | --- | --- |
| Targeted sample size | 130 | 2080 |
| extra 50% to compensate for non-response rate | 65 | 1040 |
| Required sample size | 195 | 3120 |

The sample size formula for a cross-sectional study with an anticipated proportion of positive PCR test ranging between 0.5 to 5% was used. The targeted total sample size of 2080 is expected to estimate the primary health care population PCR positivity rate (after weighting for population strata proportions) with 95% confidence and a margin of error (as a percentage of the expected estimate) ranging between 19% for the highest anticipated estimate of 5% estimate to 60% error for the lowest anticipated estimate of 0.5% for positivity rate.

## Sample size calculation for a single cross-sectional survey

To estimate a sample size for a proportion in a single cross-sectional survey, three numbers are needed:

- Estimate of the expected proportion (p)
- Desired level of absolute precision (d)
- Estimated design effect (DEFF)

The sample size formula is:

$$n=\frac{1.96^{2}p(1-p)(DEFF)}{d^{2}}$$

**References**

1. Gorstein J, Sullivan KM, Parvanta I, Begin F. Indicators and methods for cross-sectional surveys of vitamin and mineral status of populations. Micronutrient Initiative (Ottawa) and Centers for Disease Control and Prevention (Atlanta), 2007: 29.
2. Charan J, Biswas T. How to calculate sample size for different study designs in medical research?. Indian J Psychol Med. 2013;35(2):121-126. doi:10.4103/0253-7176.116232

## Sample weights calculation

Sampling weights are the inverse of the likelihood of being sampled. It’s a value is assigned to each case in the data file. The purposes of weighting at the analysis stage are:

a. To compensate for unequal probabilities of selection.

b. To compensate for non-response.

c. To adjust the weighted sample distribution for key variables of interest (for example, age, gender and nationality) to make it conform to a known population distribution.
The calculations needed are shown in table 4.

**References**

1. Yansaneh I S. Construction and use of sample weights. In: Designing Household Survey Samples: Practical Guidelines. Department of Economic and Social Affairs, Statistics Division, Studies in Methods, Series F No.98. United Nations, New York, 2005.
2. Winship C and Radbill L. Sampling Weights and Regression Analysis. Sociological Methods and Research, Sage Periodical Press, 1994: 23 (2): 230 to 257.

Table 4: Description of sample weight calculation for 16 strata based on age, gender and nationality

| **Strata** | **Nationality** | **Gender** | **Age group (years)** | **Population size** | **Targeted sample** | **Completed sample size** | **Sampling fraction** | **Wsf** | **Wres** | **WS** | **PSW** | **FWS** |
| --- | --- | --- | --- | --- | --- | --- | --- | --- | --- | --- | --- | --- |
| S1 | Non-Qatari | Female | 10-17 | 50,773 | 195 | 0 | ** | ** | ** | ** | ** | ** |
| S2 | Non-Qatari | Female | 18-39 | 238,977 | 195 | 9 | 0.00081598 | 1,225.52 | 21.6667 | 26553 | 0.4191 | 12.1069 |
| S3 | Non-Qatari | Female | 40-59 | 109,529 | 195 | 12 | 0.00178035 | 561.69 | 16.2500 | 9127 | 0.1441 | 3.1212 |
| S4 | Non-Qatari | Female | 60-74 | 12,480 | 195 | 5 | 0.015625 | 64.00 | 39.0000 | 2496 | 0.0394 | 2.0485 |
| S5 | Non-Qatari | Male | 10-17 | 54,504 | 195 | 0 | ** | ** | ** | ** | ** | ** |
| S6 | Non-Qatari | Male | 18-39 | 214,491 | 195 | 90 | 0.00090913 | 1,099.95 | 2.1667 | 2383 | 0.0376 | 0.1087 |
| S7 | Non-Qatari | Male | 40-59 | 153,540 | 195 | 70 | 0.00127003 | 787.38 | 2.7857 | 2193 | 0.0346 | 0.1286 |
| S8 | Non-Qatari | Male | 60-74 | 25,733 | 195 | 18 | 0.00757782 | 131.96 | 10.8333 | 1430 | 0.0226 | 0.3259 |
| S9 | Qatari | Female | 10-17 | 27,867 | 195 | 0 | ** | ** | ** | ** | ** | ** |
| S10 | Qatari | Female | 18-39 | 54,656 | 195 | 8 | 0.00356777 | 280.29 | 24.3750 | 6832 | 0.1078 | 3.5044 |
| S11 | Qatari | Female | 40-59 | 28,625 | 195 | 10 | 0.00681223 | 146.79 | 19.5000 | 2863 | 0.0452 | 1.1746 |
| S12 | Qatari | Female | 60-74 | 9,369 | 195 | 5 | 0.02081332 | 48.05 | 39.0000 | 1874 | 0.0296 | 1.5379 |
| S13 | Qatari | Male | 10-17 | 29,137 | 195 | 0 | ** | ** | ** | ** | ** | ** |
| S14 | Qatari | Male | 18-39 | 52,813 | 195 | 16 | 0.00369227 | 270.84 | 12.1875 | 3301 | 0.0521 | 0.8466 |
| S15 | Qatari | Male | 40-59 | 24,012 | 195 | 14 | 0.00812094 | 123.14 | 13.9286 | 1715 | 0.0271 | 0.5027 |
| S16 | Qatari | Male | 60-74 | 7,778 | 195 | 3 | 0.02507071 | 39.89 | 65.0000 | 2593 | 0.0409 | 3.5464 |
| **Total** |  |  |  | **1,094,284** | **3120** | **260** |  |  |  | **63360** | **1.0000** |  |

SF (Sampling fraction. Probability of selection for each individual in each of the sampled strata)= Population size for a specific strata / Targeted sample size for that strata

Wsf (Weight of Sampling fraction)= 1 / SF

Wres (Weight of respondents)= Targeted sample size for the specific strata / Completed sample size for that strata

WS (Weight of Strata)= Wsf x Wres

PSW (Proportional Strata Weight)= WS / Total WS

FWS (Final Weight of Strata)= (PSW * Total of completed strata sample size) / Completed Stratum sample size
